# Hippo Signaling Pathway as a Central Mediator of Receptors Tyrosine Kinases (RTKs) in Tumorigenesis

**DOI:** 10.3390/cancers12082042

**Published:** 2020-07-24

**Authors:** Taha Azad, Reza Rezaei, Abera Surendran, Ragunath Singaravelu, Stephen Boulton, Jaahnavi Dave, John C. Bell, Carolina S. Ilkow

**Affiliations:** 1 Ottawa Hospital Research Institute, Ottawa, ON K1H 8L6, Canada; tazad@ohri.ca (T.A.); reza.rezaei@uottawa.ca (R.R.); asure077@uottawa.ca (A.S.); rsingaravelu@ohri.ca (R.S.); sboulton@ohri.ca (S.B.); jdave@ohri.ca (J.D.); jbell@ohri.ca (J.C.B.); 2 Department of Biochemistry, Microbiology and Immunology, University of Ottawa, Ottawa, ON K1H 8M5, Canada

**Keywords:** Hippo signaling, receptor tyrosine kinases (RTKs), YAP, TAZ

## Abstract

The Hippo pathway plays a critical role in tissue and organ growth under normal physiological conditions, and its dysregulation in malignant growth has made it an attractive target for therapeutic intervention in the fight against cancer. To date, its complex signaling mechanisms have made it difficult to identify strong therapeutic candidates. Hippo signaling is largely carried out by two main activated signaling pathways involving receptor tyrosine kinases (RTKs)—the RTK/RAS/PI3K and the RTK-RAS-MAPK pathways. However, several RTKs have also been shown to regulate this pathway to engage downstream Hippo effectors and ultimately influence cell proliferation. In this text, we attempt to review the diverse RTK signaling pathways that influence Hippo signaling in the context of oncogenesis.

## 1. Introduction

The uncontrolled growth of cells, due to the occurrence of a variety of biological events, leads to the formation of a neoplastic tissue or a tumor. The malignant form of a tumor, a cancerous tumor, is one of the principal causes of death and has a significant effect on longevity and life expectancy [1]. Despite the internal complexity and diversity of cancers, common principles, referred to as the “hallmarks of cancers”, provide unifying themes that support research and therapeutic initiatives. Cancer development consists of 6 classic and two emerging hallmarks, including maintaining growth signaling, deactivating tumor suppressors, bypassing death signals, reaching proliferative immortality, provoking angiogenesis, stimulating invasion, and metastasis, reprogramming of energy metabolism, and escaping immune eradication [2].

The continually evolving nature and the endogenous complexity of cancer cells provide them with the ability to circumvent conventional treatment strategies. As such, the next generation of therapies are focused on a personalized approach, which may involve the simultaneous targeting of several major biological pathways contributing to the aforementioned hallmarks [3]. These novel combination therapy strategies necessitate a deeper understanding of the main signaling pathways involved in cancer progression. Hippo signaling is one of the central signaling pathways in the regulation of organ growth. This pathway also has significant involvement in cell fate decisions, maintaining stemness state of undifferentiated cells, wound healing and tissue damage response, fibrosis diseases, and tumorigenesis [4]. The direct effect of this pathway on most of the cancer hallmarks [5] makes the Hippo pathway an attractive target for cancer therapy. To better understand the role of the Hippo pathway in cancer, it is crucial to understand its interaction with other signaling pathways and the reciprocal regulatory effects between them.

There are numerous reports of external and internal signals capable of modulating the Hippo pathway, including events that influence cell polarity, cell adhesion, cell-extracellular matrix (ECM) contact and energy stress [6,7,8]. However, in the context of drug design potential, these signals are not readily targetable. Ligand-receptor mediated signaling pathways are advantageous therapeutic targets as they are easily modifiable using extracellular molecules and their modification is often more effective than downstream effectors. There are numerous methods, including molecular mimicry and antibody-mediated targeting that are effective in modulating these signaling molecules. One of the key initiators of internal signaling networks with an essential role in cell proliferation and growth is the family of receptor tyrosine kinases (RTKs). The members of this receptor family have significant effects on cancer progression in several cancer types.

The Hippo pathways connection with RTKs is not linear, and these receptors are also under the influence of the Hippo and its effectors. Due to the broad targets of Hippo effectors and their evident expression in different cellular functions, which also involve RTKs, there seems to be a multi-dimensional control mechanism between these two major cellular signaling networks. The current review aims to elaborate on this reciprocal regulatory effect.

## 2. Hippo and RTKs in Tumorigenesis

### 2.1. Hippo

**Figure 1:**
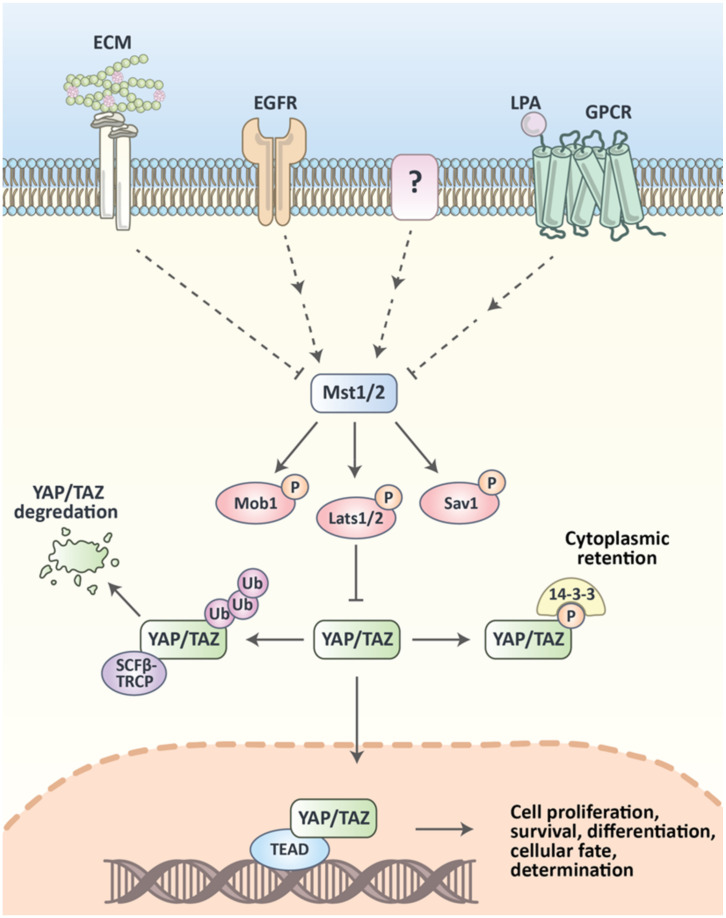
Illustration of the Hippo pathway and its regulation in mammalian cells. Yes-associated protein (YAP) and TAZ complex with TEAD can activate several cellular functions in the nucleus. The Hippo pathway function is to prevent YAP/TAZ activity by phosphorylating them, which causes their cytoplasmic retention, by 14–3–3 proteins, or their ubiquitination, by the Skp, Cullin, F-box (SCF) complex. The regulation of the Hippo pathway by upstream membrane receptors is not a comprehensively studied topic.

Even though the main components of the Hippo pathway were first discovered and studied in fruit flies, the critical role of this network in mammalian cellular signaling was soon evident. The Hippo pathway is a conserved signaling network in mammals with four main components and two main effector proteins. Except for SAV1, there are two, very closely related, proteins for each one of the pathway base proteins, including MST1 and MST2, LATS1 and LATS2, MOB1A and MOB1B; as well as YAP1 and TAZ (Figure 1). The pathway follows these steps:

- The MST1/2 phosphorylates itself and the SAV1 accessory protein
- MST1/2 in a dimerized form complexed with SAV1 will be cleaved by caspase-3 to enhance the kinase activity of the complex [9]
- The MST1/2-SAV1 complex phosphorylates MOB1A/B, which then associates with LATS1/2 [10]. It then phosphorylates LATS1/2 while connected to MOB1A/B [11].
- The phosphorylated LATS1/2-MOB1A/B will act as a kinase to phosphorylate YAP1 and TAZ [12,13]. The angiomotin proteins function in positive regulation of this reaction [14].

Phosphorylated YAP1 and TAZ either bind to 14–3–3 proteins, which sequester them in the cytosol, or will be ubiquitinated by the Skp, Cullin, F-box (SCF) complex. This eventually leads to its degradation. Unphosphorylated YAP1/TAZ interact with TEAD transcription factors, and TAZ also interacts with RUNX2 and TBX5, which activate the expression of their target genes [15,16].

**Figure 2:**
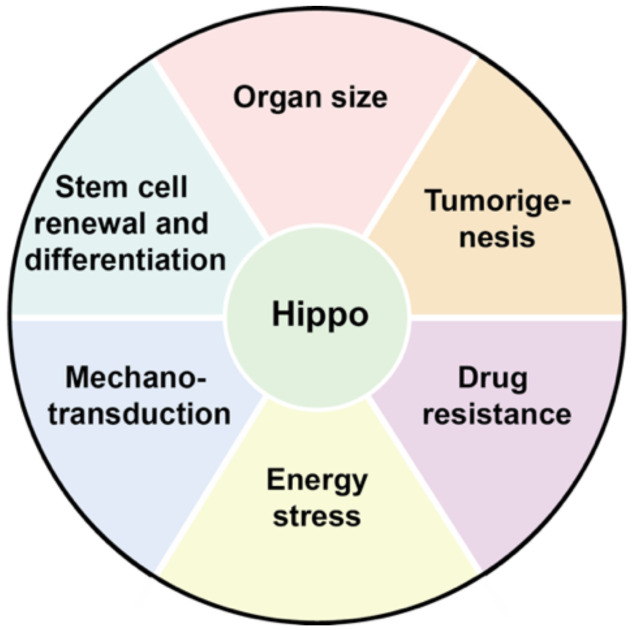
Main known biological activities regulated by the Hippo pathway. Hippo pathway and its effectors control the cell physiology by influencing several major biological functions in cells.

Therefore, the ultimate function of the Hippo pathway is the downregulation of the YAP1/TAZ target genes (e.g., *CTGF*, *CYRG1*, *NPPA*, *AREG*, etc.), which are mostly involved in cell proliferation and apoptosis [17]. The regulation of Hippo components can be a significant control point for the physiological functions which depend on the activity of these genes. The following section mentions some of these biological functions (Figure 2).

### 2.2. Physiological Functions

Many of the YAP/TAZ target genes are involved in cellular proliferation, and their inhibition by the Hippo pathway is the signal for ectodermal differentiation. Some of these targets are the master genes of stemness maintenance, such as *Nanog* and *Oct4* [18]. Hence, the high expression level of YAP is a characteristic feature of embryonic and adult stem cells [19]. The early studies on the induction of stem cell-like pluripotency state used *c-Myc*, *Sox2*, *Oct3/4*, and *KLF4* genes [20]. YAP can replace both *c-Myc* and *KLF4* to achieve the same results [21]. Significant downregulation of YAP mRNA and protein, which results in suppression of stemness genes such as *Sox2*, *PcG*, *Oct3/4*, and *Nanog*, during differentiation [22].

The direct involvement of the Hippo pathway and its effector proteins in stem cell renewal can determine the size of an organ by regulating the rate of cellular division in that organ. Hence, several regulatory proteins, mostly transmembrane proteins, interact with the Hippo pathway to distribute or interrupt proliferative signals. The effectiveness of these signals depends on the space surrounding a cell, which includes its neighboring cells and its ECM content and topology. Interactions with the environment define adhesion and polarity of a cell. Crumbs are transmembrane proteins, which in complex with WWC1, Merlin and Expanded proteins, can regulate apical-basal polarity. In Drosophila, this complex increases the activity of Yorkie, a YAP/TAZ homolog, by suppressing the Hippo pathway [23]. The cell membrane proteins, Scrib and FAT, which regulate planar cell polarity, also interact with LATS and MST proteins [24,25]. FAT senses the mechanical pressure of the ECM by linking ECM to the cytoskeleton and elevates YAP/TAZ activity based on the pressure level [7]. The interplay between different components of the ECM and transmembrane proteins and their regulatory effects on the activity of Hippo proteins can be the subject of a separate review [26]. For instance, matrix proteoglycans including Agrin and Syndecan-4 can be indicators of the mechanical situation of the environment and transmit these mechanical signals to the Hippo proteins [27,28]. Moreover, the major cellular adhesion proteins, including Angiomotin, alpha-catenin, and PTPN14, interact with Hippo components. Ultimately, all these signals influence tissue and organ growth.

Many of the YAP/TAZ target genes drive cell proliferation, a process that requires a high amount of accessible energy. Hence, there are controlling mechanisms to slow down the proliferation signals in case of a shortage of energy, glucose, or resources. Low glucose conditions mean less ATP in a cell and an elevation of the AMP/ATP ratio. This energy stress activates AMP-dependent protein kinase (AMPK), which disrupts YAP function through several mechanisms. This protein phosphorylates YAP in two positions—at one position to interrupt its interaction with TEAD [29] and at another residue to decrease its transcriptional activation function [30]. Moreover, AMPK can facilitate YAP cytoplasmic retention by LATS through its kinase activity on AMOTL1, a member of the angiomotin protein family [8,31].

Even though YAP1 activity on increasing cell proliferation and inhibiting apoptosis justifies its high level of expression and function in several cancer types, this is not the only way that YAP affects the persistence and survival of cancer cells. There are studies describing the involvement of YAP target genes in resistance against chemotherapeutic agents. The mechanism of action of anti-tubulin drugs is mediated through the activation of CDK1, which bypasses the Hippo pathway and inhibits YAP by phosphorylation at five sites. However, resistant cancer cells revive YAP1 activity by increasing mutations at these sites [32]. Resistance against Paclitaxel, an anti-tubulin chemotherapeutic drug, is achieved through induced expression of *Cyr61* and *CTGF*, both of which are YAP targets [33].

### 2.3. Connection with Tumorigenesis

Hippo proteins act as tumor suppressors that prevent the function of YAP1 and TAZ proto-oncogenes. There are numerous studies evaluating the effects of Hippo deregulation on tumorigenesis, including reports that link LATS2, and critical tumor suppressor genes, *p53* and *RB* [34,35]. Hyper-activation of YAP1/TAZ also leads to the dysregulation of other pathways involved in proliferation and tumor growth. 

The main changes to Hippo signaling in cancer occurs through modifications to gene expression or disorder in upstream regulatory signals. The rate of mutation in Hippo effectors, YAP1/TAZ, is negligible; however, modifications in their expression patterns is a common phenomenon in several cancer types. Some reports recount the upregulation of the YAP1/TAZ in the head, neck, and gynecologic cancers [36]. Moreover, between more than thirty cancer types, the gene expression profile of squamous cell carcinomas (SCCs) had the highest level of YAP1/TAZ expression [37].

The main Hippo components are differentially expressed across cancer types, such as in the cases of MST1/2 in soft tissue sarcomas [38,39,40], LATS1/2 in astrocytoma and breast cancers [41,42], TAZ in breast cancer [43,44], and MOB1 in lung and colon cancers [45,46].

As noted, one of the main ways the Hippo pathway influences tumorigenesis is by altering the activity of other cascades. Tumor suppressor gene *NF2* is highly susceptible to mutations, and its protein product, Merlin, has a direct activatory effect on Hippo. Hence, Merlin’s activity can lead to the downregulation of the YAP1/TAZ targets. *RAS* genes are molecular switches with critical regulatory effects on many signaling networks. YAP partly controls the transcription level of these genes. Consequently, the inactivation of Merlin can lead to *RAS* expression due to the formation of the YAP-TEAD complex. Verteporfin is a drug capable of inhibiting YAP-TEAD, which could reverse the effects of Merlin inactivation [47].

Targeting YAP in regulatory T cells (Treg) can disrupt the activity of these cells to promote antitumor immune responses. Treg cells suppress immune activation, and FOXP3 is a critical transcription factor for the function of these cells. By targeting YAP, which has been demonstrated to enhance FOXP3 expression, we can attempt to control tumor growth [48].

During the process of LATS1/2 activation, kibra (WWC1) binds LATS proteins and promotes their function, which causes YAP1/TAZ cytoplasmic localization [49]. The ACTL6A, a subunit of the chromatin-remodeling complex, and the tumor protein p63, are highly amplified in head and neck squamous cell carcinoma (HNSCC). The interaction of these two proteins alters the epigenetic state of WWC1 and directly represses its expression and the activity of Hippo proteins. This ultimately results in YAP mediated proliferation and tumorigenesis [47].

Even though the primary mechanism of YAP hyper-activation is through the dysregulation of the canonical Hippo components, some proteins bypass the Hippo pathway and directly activate YAP. The gain of function mutation in one set of such proteins is in the Gαq family members. It is significantly frequent in uveal melanoma, occurring in approximately 83% of these melanomas. Gαq and Trio, the guanine exchange factor for Gαq, along with RhoA and Rac1 GTPases, initiate a signaling network that promotes YAP activation and leads to YAP-dependent growth and proliferation of cancer cells independent of other Hippo pathway proteins [50].

The examples above highlight the interplay between Hippo proteins and some regulatory proteins on the same level or downstream of the pathway. However, there are several upstream regulators of this signaling cascade, including receptor tyrosine kinases, which can inhibit the initiation of the pathway and increase proliferation signals. The regulatory effects of these proteins on the Hippo pathway, are poorly understood.

## 3. RTKs

Protein phosphorylation is one of the most common ways of altering a protein’s function and protein kinases are major players in signaling networks. Tyrosine kinases are switches that can stimulate or deactivate many cellular functions. Even though there are cytoplasmic tyrosine kinases, the membrane-associated RTKs are crucial in the activation state of signaling pathways due to their direct contact with the external environment [51]. The human genome encodes 58 different RTKs that are involved in many signaling cascades [52]. Epidermal growth factor receptor (EGFR), insulin receptor (IR), platelet-derived growth factor receptor (PDGFR), and several other physiologically important receptors are in this family and their connection with the Hippo pathway will be discussed in the following sections.

**Figure 3:**
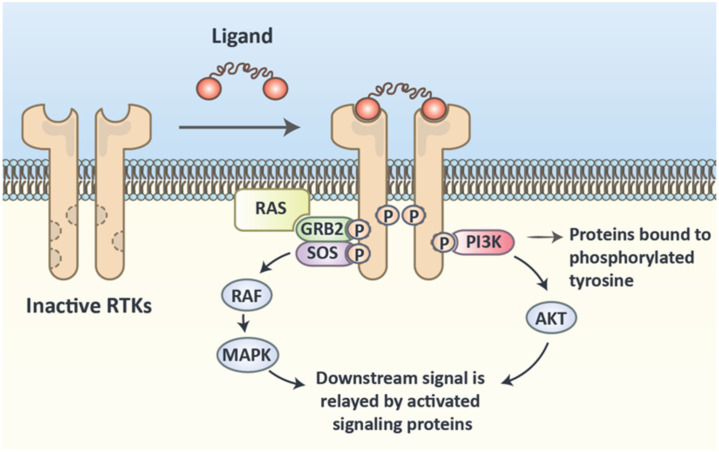
Receptor Tyrosine Kinase (RTK) domains and activation process. This schematic representation depicts the dimerization process of RTKs following activation by their specific ligands. A series of self-phosphorylation and phosphorylation of proteins linked to the intracellular protein binding domain of RTKs initiates downstream signaling cascades.

The overall structure of RTKs is composed of an external ligand-binding domain, a transmembrane domain, and intracellular catalytic and adaptor domains. The conformational change after binding a ligand leads to dimerization and autophosphorylation of monomers [53]. The tyrosine residues in the adaptor part of the receptor will be phosphorylated by catalytic domains. Effector proteins can land on the RTK’s adaptor region and be phosphorylated, which starts a cascade (Figure 3).

### 3.1. RTK/RAS/PI3K and RTK-RAS-MAPK Are Two Main Activated Signaling Pathways After Ligand Binding

Tyrosine phosphorylation by RTKs is important for the recruitment and activation of a variety of signaling proteins. Most RTK tyrosine phosphorylation sites are located in non-catalytic regions of the receptor molecule. RTKs should be considered as a platform for the recognition and recruitment of a specific complement of signaling proteins [54]. SH2 (Src homology 2) and PTB (phosphotyrosine binding) are protein domains that evolved during metazoan evolution to recognize the tyrosine phosphorylation sites of other proteins such as RTKs. Grb2 is an adaptor protein with an SH2 domain located at its C-terminus. When RTKs are activated, Grb2 binds directly to RTKs and recruits a guanine nucleotide exchange factor (GEF), known as Son of Sevenless (SoS). This recruitment activates SoS, which then exchanges GDP with GTP in RAS. RAS-GDP will be fully active when its nucleotide is replaced with GTP. GTP hydrolysis by RAS then triggers the MAPK pathway by activation of RAF kinase. Grb2 associates with Gab1, another adaptor protein, and triggers phosphoinositide 3-kinase (PI3K)–Akt pathway through RAS [55]. Nearly all RTKs use this event to activate PI3K and MAPK pathways through RAS activation. There is an increasing number of publications showing several links between the RAS, PI3K, and MAPK kinase pathways with the Hippo signaling pathway.

### 3.2. YAP Mediates Main Functions of the Oncogene RAS

Cancer cells require the continuous expression of RAS during tumor progression. This addiction to the RAS oncogene is due to the activation of its downstream pathways, mainly the PI3K and MAPK pathways. Shao et al. performed a systematic screen using 15,294 open reading frames in a human RAS-dependent cancer cell line designed to express an inducible RAS-specific shRNA [56]. Interestingly, they found that YAP can rescue cell viability upon suppression of RAS. This resistance to RAS suppression was confirmed in several models including in lung and colon cancer. Since RAS signaling activates mainly the PI3K and MAPK pathway, different inhibitors for these two pathways were tested. YAP was able to rescue cancer cells from RAS suppression even after inhibition of the PI3K and MAPK pathways. Surprisingly, it was also observed that the top gene set in YAP and RAS transcription signature as well as epithelial to mesenchymal transition (EMT) signatures were significantly overlapping, indicating the transcriptional regulation of EMT by YAP as a significant component of RAS signaling [56].

Another study demonstrated that the upregulation of YAP can bypass the requirement for oncogenic RAS in anchorage-independent growth in vitro and for tumor formation in vivo [57]. Consistently, activation of the Hippo pathway and degradation of YAP via the TrCP-SCF ubiquitin ligase complex prevented RAS-mediated cellular transformation and tumor formation. YAP can form a positive feedback loop with RAS. For example, Amphiregulin, a YAP-TEAD transcription target [57], can activate RAS through EGFR, resulting in more YAP activation and full transformation of EGFR positive cell lines. 

For cancer types with frequent mutations in RAS, such as pancreatic ductal adenocarcinoma (PDAC), there is evidence that YAP mRNA and protein levels are increased in human PDAC relative to normal pancreatic tissues [58]. In genetically engineered mice with constitutively active RAS, tissue-specific YAP deletion can prevent the formation of early neoplastic lesions without affecting normal pancreatic endocrine function. 

### 3.3. YAP is a Downstream Effector of the MAPK Pathway

Activation of RTK initiates MAPK pathway signaling through RAS GTPase activity. The best-known effectors of RAS are RAF and phosphatidylinositol-3-kinase (PI3K) [59]. The RAS-RAF complex is translocated to the cell membrane, where RAS GTP activates serine/threonine kinase activity of RAF. Activated RAF phosphorylates MEK, which then phosphorylates and activates ERK. Activated ERK phosphorylates various targets, including proteins involved in cell cycle regulation such as cyclin-dependent kinases (CDKs) [60]. The mutation rate in RAF is 50–70% among all melanoma cancers and 80% of these cases are represented by a single mutation (V600E) which causes a 10-fold increase of RAF kinase activity [61]. This type of mutation is also reported in other malignancies such as lung cancer [61], colorectal cancer [62], cholangiocarcinoma [63], and gastric cancer [64]. Similar to many other developed inhibitors, RAF inhibitors showed resistance after clinical trials. Thus, it is very important to find a key effector, downstream of the MAPK pathway as an alternative therapeutic target.

Immunoprecipitation studies in melanoma cancer cell lines (C32, HS695T, SK-MEL-28, and A375) have shown the interaction between RAF and MST [65]. Knocking down RAF results in YAP and TAZ protein reduction. The RAF interaction domain is located near the transphosphorylation site of MST. RAF prevents MST activation by interfering with its dimerization and trans-phosphorylation [66]. Suppression of MST in human and mouse RAF knock out cell lines significantly reduced apoptosis. Conversely, depletion of RAF in wild type cells shows the opposite phenotype. Combining mathematical modeling with experimental validation revealed novel negative feedback in which LATS1/2 can phosphorylate RAF on Ser 259. This feedback regulation inhibits MST suppression by RAF, causing Hippo pathway activation and finally YAP/TAZ cytoplasm retention [67]. You et al. reported another novel mechanism for regulation of the Hippo pathway by the MAPK pathway [68], where inhibition of ERK, either by small interfering RNA (siRNA) or added inhibitors, reduced YAP protein levels as well as downstream expression of Hippo genes such as *CTGF* and *BIRC5*. However, YAP mRNA levels were not affected, implying that YAP protein stability is regulated by ERK. Several studies show a potential link between the Hippo and MAPK pathways [69,70]; however, further investigation is required for determining the potential mechanism of this crosstalk.

### 3.4. YAP is a Downstream Effector of the PI3K Pathway

When PI3K is activated by RAS, it phosphorylates phosphatidylinositol-4,5-bisphosphate (PIP2) to phosphatidylinositol-3,4,5-triphosphate (PIP3) which then activates AKT (protein kinase B). This activation plays important roles in cell proliferation, cytoskeletal rearrangement, and tumor progression [59]. The PI3K protein includes regulatory and catalytic subunits. The catalytic subunit includes three different isoforms: PI3KCA, PI3KCB, and PI3KCD [71]. Alterations to PI3KCA such as mutations, amplifications, and fusions occur in 55% of head and neck squamous cell carcinomas (HNSCC) and are associated with poor prognosis. Escudero et al. performed whole-genome expression profiling in primary HNSCC tumors from The Cancer Genome Atlas (TCGA) and evaluated the relationship of PI3KCA expression with nuclear YAP in tissue microarrays [72]. This study showed that YAP was significantly localized in the nucleus in highly expressed PI3KCA samples. 

Recently, we performed a systematic gain-of-function screen for kinases involved in mammary tumorigenesis. In this screen, we made a kinase overexpressing library (KOL) and established a heterogeneous population of nontumorigenic mammary epithelial cells infected with this KOL library. Following transformation assays, mass spectrometry was performed on positive colonies and it was found that PI3KCB is a transformation-inducing kinase in breast cells [73]. We showed PI3KCB induces transformation and reduces cell death through YAP and TAZ activation. Mechanistically, we showed YAP/TAZ activation occurs through multiple signaling pathways including LATS-dependent and LATS independent pathways.

PI3K and the Hippo pathways also interact through MST and AKT. Activated AKT phosphorylates MST on T117 and T384. This phosphorylation limits MST interactions, including MST dimerization, transphosphorylation, and binding to the scaffold protein, RASSF1A [74]. 

Interestingly, YAP forms a positive feedback loop with the PI3K pathway. Analysis of differential gene expression for YAP gain- or loss-of-function with genome-wide identification of YAP-bound loci showed that PI3KCB is a YAP-TEAD transcription target that is sufficient to activate the PI3K pathway and is necessary for cardiomyocyte proliferation and survival [75]. The role of this feedback loop in the context of tumorigenesis is not yet clear. Phosphatase and tensin homolog (PTEN) is another known regulator of the PI3K and Hippo pathways. Gastric cancer cells show more cell proliferation and tumor formation when PTEN is inactivated [76]. Immunohistochemical analysis of gastric cancer tissue showed a significant correlation between YAP nuclear localization and phosphorylated PTEN. Mechanistically, inactivated PTEN results in inhibition of LATS activation through preventing its interaction with MOB1. 

In summary, two main downstream signaling pathways of RTKs, the MAPK, and PI3K pathways, have been demonstrated to regulate the Hippo signaling pathway. 

## 4. Several RTKs Regulate the Hippo Pathway

### 4.1. EGFR and the Hippo Pathway Regulate Each Other through a Positive Feedback Loop

The detailed mechanisms of Hippo pathway regulation by EGFR was determined through studies of Drosophila. EGFR regulates Yorkie nuclear localization and Yorkie is necessary for EGFR-induced cell proliferation in Drosophila [77]. Ajuba, known as a negative regulator of LATS, is phosphorylated by ERK after activation of RAS-MAPK by EGFR. Phosphorylated Ajuba then interacts with the Sav/LATS complex and inhibits LATS kinase activity. This phenotype can be suppressed using MEK or ERK inhibitors. The EGFR-RAS-MAPK and Ajuba interaction is also conserved in mammals and regulates LATS activity and YAP nuclear localization. Fan et al. reported another mechanism for regulating Hippo by EGFR [78]. They showed rapid YAP nuclear localization and LATS inhibition after EGF treatment by using a small molecule inhibitor screen of downstream effector pathways. They identified that the EGF receptor inhibits the Hippo pathway through activation of PI3K and phosphoinositide-dependent kinase (PDK1), and this was independent of AKT activity. PDK1 interacts with LATS and inhibits it through a scaffold protein, Salvador. Similarly, in another study, it was shown that the EGFR-PI3K-PDK1 pathway regulates YAP activation in hepatocellular carcinoma [79].

EGFR is highly expressed in epithelial and fibroblast cells. The EGFR family includes four members, ERBB1-4. All members have a glycosylated extracellular domain, a single hydrophobic transmembrane domain, and an intracellular domain with kinase activity [80]. Among seven known ligands, all of them bind ERBB1 and ERBB4, two bind ERBB3, and none bind ERBB2. EGFR/ERBB1 ligands include epidermal growth factor (EGF), transforming growth factor-alpha (TGF-α), amphiregulin, betacellulin, epigen, epiregulin, heparin-binding EGF, and neuregulin 2β [81]. EGFR antibodies and EGFR tyrosine kinase inhibitors have been explored in many clinical trials. Although the initial responses were promising, in most cases, the tumors recurred faster and were more aggressive [82]. Several studies have revealed different mechanisms for acquiring this resistance. For example, EGFR in-frame mutations can constitutively activate the receptor, making anti-EGFR antibodies ineffective for tumor suppression. Several mutations such as T790M hinder the kinase inhibitor binding site, resulting in EGFR inhibitor resistance [82]. Therefore, it is important to find an alternative target for single or combinational therapy in EGFR positive patients. 

In general, resistance to EGFR kinase inhibitors can be divided into two categories including primary resistance, which relies on other oncogenes such as *RAS*, or acquired resistance, which depends on new mutations. Investigations of gefitinib-resistant A549 and PC9 cell lines compared with parental cells showed that YAP can play important roles in both primary and acquired resistance [83]. Interestingly, YAP inhibition by chemical inhibition or siRNA knockdown can restore EGFR kinase inhibitor sensitivity in lung cancer cell lines. EGFR overexpression is one mechanism of acquired resistance. It has been reported that YAP-TEAD can positively regulate sustained EGFR expression in esophageal cancer (EC) [84]. Consistently, YAP inhibition by Verteporfin restores esophageal cancer cell sensitivity to 5-FU and docetaxel cytotoxins. Lee et al. demonstrated that YAP plays an important role in EGFR kinase inhibitor resistance in lung adenocarcinomas [85]. Similarly, YAP inhibition can restore EGFR kinase inhibitor sensitivity. The T790M mutation is more frequently associated with acquired resistance to an EGFR inhibitor in lung cancer patients. Furthermore, non-small cell lung cancer (NSCLC) cells have a high level of TAZ expression, which when suppressed in T790M drug-resistant cells, reduces anchorage-independent growth in vitro and tumor formation and resistance to gefitinib in vivo [86].

He et al. showed a positive signaling loop between EGFR and the Hippo signaling pathway [87]. YAP upregulates transforming growth factor-alpha (TGF-α), amphiregulin, and EGFR expression. Upregulation of EGFR with its ligands then inhibits the Hippo pathway and activates YAP to induce cervical cancer cell proliferation, migration, and anchorage-independent cell growth. The trigger of this positive signaling loop could be the Human Papillomavirus E6 protein (HPV E6), a major etiological player in cervical cancer. HPV E6 increases YAP stability and protein levels by preventing proteasome-dependent YAP degradation. This in turn triggers the positive signaling loop between Hippo and EGFR signaling pathways [87]. The positive feedback has been also reported in diabetic kidney patients whereby EGFR signaling in renal epithelial cells can exacerbate diabetic kidney injury through YAP activation [88].

ErBb2, also known as Human epidermal growth factor receptor 2 (HER2), is highly expressed in 15–20% of breast cancers. As ERBB2 (HER2) does not have any known ligand, it needs to interact with other EGFR family member proteins to be activated by transphosphorylation. Lapatinib is a potent inhibitor for both EGFR and HER2, and good responses are achieved for HER2 positive patients. Unfortunately, tumor regrowth is typically observed due to drug resistance [89]. Lapatinib resistance can be eliminated by knocking down YAP and TAZ or through pharmacological inhibition of YAP-TEAD in vitro. In addition, YAP suppression slowed the growth of implanted HER2-amplified tumors in vivo [90]. This further supports the use of combinational therapies, including the inhibition of Hippo pathway components, to overcome EGFR inhibitor resistance.

YAP has been reported to interacts with the carboxyl-terminal PPXY motif of ERBB4 through its WW domain [91]. ERBB4 is a unique member of the EGFR family that can be proteolytically cleaved after Neuregulin or 12-O-tetradecanoylphorbol-13-acetate treatment. YAP and the cleaved fragment of ERBB4 translocate into the nucleus, after which the co-localization significantly increases the co-transcriptional activity of YAP [92]. Interestingly, ERBB4 forms a complex with YAP and TEAD, which is independent of YAP as demonstrated by a YAP binding-deficient mutant of TEAD1 (Y406A) that efficiently interacted with ERBB4. A DOX inducible YAP knockdown showed a critical role for YAP-mediated migration when ERBB4 was activated. Moreover, it has been shown that the ERBB4-YAP interaction promotes trastuzumab resistance in HER2-positive gastric cancer by inducing epithelial to mesenchymal transition [93].

### 4.2. PDGFR Regulates YAP Activity through Src Family Kinases

Cholangiocarcinoma is an aggressive malignancy with limited treatment options, as chemotherapies only increase survival modestly [94]. Platelet-derived growth factor receptor (PDGFR) and its ligand are highly expressed in cholangiocarcinoma. YAP is also highly expressed and mainly localized in the nucleus. Smoot et al. studied the potential regulation of YAP by PDGFR [95]. They confirmed YAP nuclear localization upon PDGFR activation in human, mouse, and cholangiocarcinoma cell lines. PDGFR pharmacologic inhibition increased YAP cytoplasmic retention and reduced the expression of YAP target genes such as CTGF and Cyr61. Interestingly, it was revealed that PDGFR activates Src family kinases (SFKs) to interact with and phosphorylate YAP on Tyrosine 407. This tyrosine phosphorylation activates YAP independently of canonical serine 127 phosphorylation. MCL-1 is one of the main YAP target genes that is increased upon tyrosine phosphorylation, and this event facilitates increased cell viability and tumor growth, both in vitro and in vivo [95]. Similar to several other RTKs, the Hippo and PDGFR signaling pathways can form a positive feedback loop, such as in malignant peripheral nerve sheath tumors (MPNSTs). LATS deficiency causes YAP and TAZ hyperactivation, which in turn induces the transformation of Schwann cells (SC) to MPNST [96]. Genome-wide profiling showed that YAP-TEAD activates the PDGFR pathway through increasing its expression and activating the RAS signaling pathway.

### 4.3. Interaction of Insulin Signaling and the Hippo Pathway

Insulin/insulin growth factor receptor signaling is one of the main regulators of cell metabolism, cell proliferation, and tissue growth. Insulin signaling activates Yorkie in Drosophila to induce cell proliferation [97]. Insulin signaling activates PI3K and interacts with the Hippo pathway in Drosophila through PDK1, which inhibits Warts activity and increases Yorkie nuclear localization. Yorkie inhibition disables the insulin pathway and prevents the induction of cell proliferation [97]. In human cell lines, it has been reported that treating PDAC cells such as PANC-1 and MiaPaCa-2 with insulin reduces YAP-pS127. This results in its nuclear localization and expression of YAP/TEAD–regulated genes such as *CTGF*, *Cyr61*, and *CXCL5* [98]. The use of siRNAs and inhibitors against signaling components downstream of the insulin receptor has confirmed the regulation of the Hippo pathway through PI3K and PDK1.

Recently it has been reported that insulin receptor substrate 1 (IRS1) in breast cancer cells is a transcriptional target of TAZ [99]. YAP-TEAD interacts with the promoter region of IRS1. TAZ deregulation in breast cancer causes IRS1 overexpression, which amplifies the insulin response and increases cell proliferation in a 3-dimensional Matrigel culture. TAZ also regulates the expression of IRS2 in hepatic steatosis and liver cancer [100]. Induced expression of IRS2 by high YAP/TAZ levels activates PI3K-AKT signaling and this positive feedback loop can be turned off by combining siYAP/siTAZ and metformin [101].

These findings serve as another example of a positive feedback loop relationship between the Hippo pathway and RTKs.

### 4.4. NTRK Is a Positive Regulator of YAP

Yang et al. showed that nerve growth factor (NGF) receptor tyrosine kinase (NTRK1) regulates YAP activity through the Hippo pathway [102]. They co-transfected a luciferase reporter driven by the CTGF promoter and YAP into HEK293 cells and screened for novel kinase inhibitors regulating the Hippo pathway. In their screen, Ro 08-2750, which blocks NGF binding to NTRK1, suppressed the reporter by more than 80%. NTRK1 inhibition with Ro 08-2750 reduced cell proliferation and migration in several cells including PANC1 and MDA-MB231 cells. It was also shown that NTRK1 inhibition activated LATS and increased YAP-pS127 and YAP cytoplasmic retention. Notably, YAP-S127A (the active form of YAP) was able to eliminate the Ro 08-2750 effect in cell migration assays.

### 4.5. c-MET Regulates HIF-1 and EMT through the Hippo Pathway

With less than a 7% five-year survival rate, PDAC has emerged as one of the most aggressive cancers [103]. Pancreatic stellate cells (PSCs) are an important component of the tumor microenvironment in PDAC. One of the main factors secreted by PSCs is the hepatocyte growth factor (HGF). MET receptor tyrosine kinase and its ligand, HGF, are involved in the regulation of tissue homeostasis under normal physiological conditions. HGF is a pleiotropic factor secreted in an inactive form and requires cleavage by extracellular proteases to make it active. During embryogenesis, the HGF/MET axis regulates the migration of many progenitor cells of different tissue types such as skeletal muscle [104]. Dysregulation of MET in several cancers occurs due to its overexpression, overexpression of HGF, activating mutations, or an autocrine/paracrine/endocrine loop dysregulation [105]. In several PDAC cell lines such as PANC-1 and MiaPaCa-2, HGF causes YAP nuclear localization [106]. Interestingly, YAP interacts with HIF-1 upon HGF treatment and induces cancer stem cell pluripotency via increasing Nanog, OCT4, and SOX-2 expression. The HGF/MET/YAP/HIF-1α axis induces hexokinase 2 expression to support glycolytic metabolism in the tumor [106].

HGF/MET regulates epithelial to mesenchymal transition (EMT) in normal physiology as well as during cancer progression [107]. HGF induces EMT in several highly expressing MET cell lines such as in Madin-Darby kidney epithelial (MDCK) cells. Farrell et al. performed protein expression profiling using mass spectrometry after treating MDCK cells with HGF [108]. Following HGF treatment, ITCH ubiquitin ligase expression increased significantly. They showed HGF induced EMT in MDCK cells by inhibiting the Hippo pathway via ITCH. ITCH ubiquitin ligase regulates LATS degradation and is overexpressed in a broad spectrum of human cancers [109].

### 4.6. Receptor Tyrosine Kinase Epha2 Regulates YAP/TAZ through Gtpase Rho and ROCK

Eph receptors are the largest family of RTKs, containing more than 14 different RTKs. Eph receptors are involved in a wide variety of biological functions such as cell attachment, cell shape, cell mobility, angiogenesis, and tumorigenesis [110,111]. There are two types of ligands for Eph receptors, Ephrin-As and Ephrin-Bs. Ephrin-As are glycosyl-phosphatidyl-inositol anchored and Ephrin-Bs are ligands with short transmembrane and cytoplasmic domains. Eph receptors and their ligands form a bi-directional signaling pathway in both receptor and ligand presenting cells. Eph receptors and Ephrins play an important role in tumor progression through poorly understood mechanisms [112].

Glutamine addiction is frequently observed in many cancers [113]. Glutamine is a non-essential amino acid that can be produced from glucose in normal cells; however, many cancer cells need to uptake large amounts of glutamine to maintain mitochondrial membrane integrity as well as NADH balance. Receptor tyrosine kinase EphA2 activates YAP/TAZ-TEAD to promote glutamine metabolism in vitro and in vivo [114]. Inhibiting YAP/TAZ reduces the expression of important genes involved in glutamine uptake and metabolism, such as *SLC1A5* and *GLS*. Both of these genes possess several TEAD binding sites in their promoter region and interact physically with YAP/TAZ through TEAD. Inhibition studies across various points in the pathway, and confirmation of the results with complementary approaches have shown that receptor EphA2 regulates YAP/TAZ through Rho GTPase and ROCK [114]. In another study, Mohseni et al. developed an improved transcriptional reporter containing 14 copies of the known TEAD DNA-binding sequence and performed an RNAi-based-kinome screen using this reporter. Their screen and preliminary validation showed several Eph receptors including EphA7, EphA4-6 and EphA8 regulated YAP activity [115]. Although this finding is very interesting, additional studies are needed to determine the precise mechanisms of the interactions.

Interestingly, YAP/TAZ also plays important roles in reverse signaling in ligand presenting cells. For example, EphB2 expressing cells in bone marrow interact physically with TAZ. Upon activation, the cytoplasmic domain of EphB2 and TAZ co-localize into the nucleus and regulate the transcription of several important genes involved in bone marrow stromal cell differentiation and bone formation [116].

### 4.7. Receptor Tyrosine Kinases VEGFR, Tie and FGFR Regulate Angiogenesis through YAP/TAZ

Blood vessel formation, or angiogenesis, is a dynamic process involving cell migration, proliferation, and differentiation. The Hippo pathway’s critical role in these physiological processes indicates the importance of this pathway in angiogenesis [110]. VEGF receptors and Tie receptors, two subfamilies of RTKs widely expressed in endothelial cells, are the building blocks of blood vessels and are involved in angiogenesis signaling. Moreover, there are reports on the regulation of angiogenesis by other RTKs, such as fibroblast growth factor receptors (FGFRs) and platelet-derived growth factor receptors (PDGFRs). Even though the mechanisms controlling interactions between these signaling molecules and their precise role in angiogenesis is not fully elucidated, the interaction of all these RTKs with the Hippo pathway is evident. The relationship between these ligand/receptors and Hippo is reciprocal. RTKs regulate Hippo components and YAP/TAZ activity, and YAP/TAZ target genes expression affects the activity and expression of RTKs.

In a recent study involving a biosensor monitoring LATS kinase activity [117], significant suppression of LATS was observed after VEGF treatment. Further investigation of the mechanism underlying this suppression revealed that inhibition of the Hippo by VEGF/VEGFR occurs through reduced phosphorylation of MST1/2 by PI3k/MAPK pathway [118]. Moreover, this study showed that YAP/TAZ knockdown significantly suppresses VEGF/-induced angiogenesis by an in vitro tube formation assay. This knockdown dramatically decreased Angiopoietin-2 (ANG-2) and CYR61 expression, which promotes angiogenesis.

In a similar study, Wang et al. showed that VEGF/VEGFR2 activate YAP/TAZ by regulating actin cytoskeleton dynamics. They found SFKs and Rho GTPases to be responsible for this effect on YAP/TAZ by decreasing LATS phosphorylation [119]. Moreover, they showed that the absence of YAP/TAZ leads to impaired cytoskeletal rearrangements and subsequent problems in VEGFR2 trafficking from the Golgi to the plasma membrane. These effects alter the cellular distribution of VEGFR2 and reduce cell migration, compromising the process of angiogenesis.

The second important set of receptors involved in angiogenesis are Tie1-Tie2 receptors and their Angiopoietin1-2 (ANG1-2) ligands. Studies show that Tie2 is the functional receptor, and Tie1 can attenuate Tie2 homodimer signaling by making Tie1-Tie2 heterodimers [120]. Moreover, ANG2 is both an antagonist of ANG1 and also a weak agonist in the absence of ANG-1. A theoretical hypothesis claims that the role of ANG-2 is to trigger angiogenesis by destabilizing vessels after VEGF signaling and ANG-1 is active in maintaining angiogenic signals as the process proceeds to completion.

The regulation of ANG-2 gene expression by YAP1 has been confirmed in several studies [118,119,121,122]. One of these studies also proposed STAT3 as a novel transcriptional partner of YAP1 in the nucleus. YAP1/STAT3 complex regulates ANG-2 expression, promoting angiogenesis in the postnatal retina and tumor tissues [121]. Moreover, YAP1 knockdown regulates Tie2 expression, such that knocking down YAP1 dramatically decreases Tie2 expression. Lower Tie2 expression blocks angiogenesis and epithelial budding formation in vitro and inhibits compensatory lung growth and vascular formation in vivo [123]. Another study compared YAP/ANG-2 signaling effect on endothelial cell (EC) proliferation in different confluency conditions. Their results highlight the role of VE-cadherin-mediated EC contact on YAP activity and localization through PI3K-Akt activation. Increased EC density correlates with more VE-cadherin and less YAP activity. Hence, YAP affects ANG-2 expression in sparse cells much more than confluent cells and YAP knockdown causes decreased vascular density with less branching points [122].

Apart from the principal VEGFRs and Tie-ANG receptors, fibroblast growth factor receptors (FGFRs) are also involved in the process of angiogenesis. These receptors participate in several other physiological phenomena including growth, wound healing, and embryogenesis. Members of the FGFR family of RTKs and their ligands interact with the Hippo pathway both in normal and tumor cells. In one study on osteoblastic MC3T3-E1 cells, FGF2 treatment led to decreased levels of the TAZ protein and this effect was correlated with SAPK/JNK MAP kinase activity [124]. Another study determined an autocrine/paracrine-positive feedback loop between FGF2 and YAP, and suggested that this signaling drives the progression of the high-grade serous carcinoma from fallopian tube secretory ECs [125]. 

Rizvi et al. explored an autocrine Hippo/FGFR signaling in cholangiocarcinoma and determined that FGFR1, −2, and −4 expression is in part driven by YAP/TBX5 complex, but this is not true for FGFR3 because it doesn’t have a TBX5 binding sequence. Interestingly, YAP activity is triggered largely by FGF5/FGFR2 activation. The FGF5/FGFR2 effect on YAP is mediated by reducing LATS cellular levels to prevent YAP phosphorylation [126]. FGFR3 and Hippo are linked by the ETV5 protein in bladder cancer cells. FGF1/FGFR3 and MAPK/ERK-mediated increase of ETV5 levels lead to elevated TAZ activity [127]. In lens epithelial cells, FGF treatment affects total YAP expression and activity differentially in a dose-dependent fashion, such that lower doses of FGF treatment increases total YAP expression but not the expression of core Hippo components, thereby triggering lens cell proliferation. However, higher doses greatly increase Hippo expression and cause YAP phosphorylation, triggering lens fiber differentiation [128]. Another study investigating the mechanism underlying breast cancer cell resistance to MST1/2-dependent apoptosis showed that FGFR4 phosphorylates MST1/2 and inhibits its activation [129].

### 4.8. FGFR, RET, and MERTK Can Bypass the Hippo Pathway by Direct Phosphorylation of YAP/TAZ

**Figure 4:**
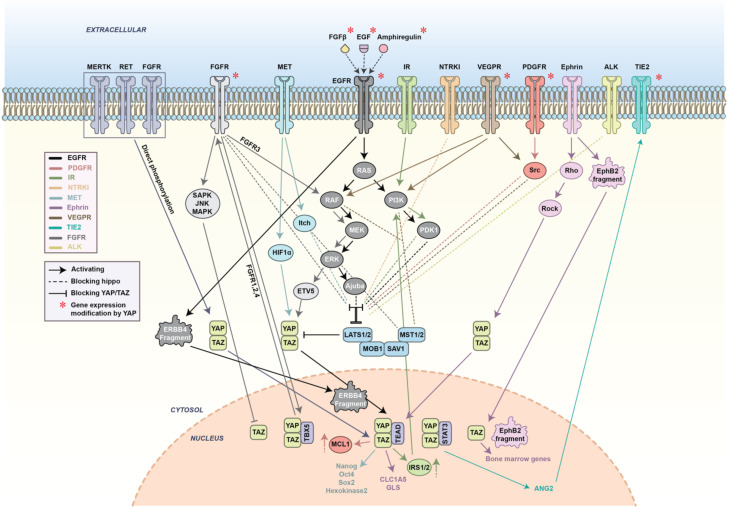
The network of known interactions between RTKs and the Hippo pathway. RTKs can directly phosphorylate, indirectly regulate, and colocalize in the nucleus with YAP/TAZ. YAP/TAZ can affect RTKs by changing the expression level of these receptors or through activation of their target genes. The interplay between Hippo and RTKs has been linked to various cancer types.

Even though most of the interactions between RTKs and Hippo occur through PI3K/Akt and MAPK signaling cascades, there are some reports on the direct interaction between ERBB4 and YAP [92] (Figure 4). In one of our recent investigations, we confirmed the existence of such interactions between FGFR, RET, MERTK receptors, and YAP. Firstly, we showed that FGFR and YAP are closely localized in the cytoplasm of BHE cells and following FGF treatment, they colocalize in the nucleus. Moreover, we determined specific tyrosine residues on YAP which are directly phosphorylated by FGFR, and except for an FGFR inhibitor, no other protein inhibitors of the ones tested could reverse this effect. Further investigation should serve to determine whether RET and MERTK receptors have similar effects on YAP and whether they can directly phosphorylate this protein. Searching for the physiological significance of these phosphorylation events, we found that phosphorylated YAP/TAZ is triggered to mediate FGF/GDNF/Gas6-induced tumorigenesis and metastasis and starts a positive feedback loop [130].

### 4.9. Anaplastic Lymphoma Kinase (ALK), a Novel Activatory Relationship with YAP/TAZ

The aforementioned examples are not the only instances of interconnecting signaling networks between the Hippo pathway and RTKs, as numerous active studies are investigating this relationship. For instance, a recent study uses a novel biosensor [131] to investigate possible regulation of the Hippo by Anaplastic lymphoma kinase (ALK) receptor [132]. ALK is an RTK with notable mRNA expression in the human brain and some other organs [133]. There are traces of ALK in several cancer types including neuroblastoma, lung cancer, and anaplastic large cell lymphoma, making it a viable therapeutic target [134]. However, there is little information, except for the bodies of work mentioned in this review, on the connection between the Hippo pathway and ALK. This study is one of the first to confirm the role of overexpressed ALK in the induction of tumorigenesis and proposes that YAP activation following LATS inhibition by ALK could have a significant effect on mediating this phenotype [132] (Table 1).

## 5. Conclusions

Originally identified as a key player in physiological development, we have now come to appreciate the broader influence of the Hippo pathway on numerous cellular processes, including in malignancies such as cancer. The intersection of the Hippo pathway with RTKs, master regulators of cell signaling, is unsurprising as Hippo appears to have a seemingly ubiquitous influence across the cell. While Hippo’s main effects are executed through the RTK/RAS/PI3K and RTK-RAS-MAPK signaling pathways, we have highlighted that diverse molecular interactions contribute to the ultimate downregulation of YAP1/TAZ target genes. Continued investigation to better understand this complex signaling pathway may provide the coveted therapeutic targets that will effectively intervene upstream signaling events in the tumor microenvironment.

## Figures and Tables

**Table 1:** Summary of the reciprocal regulation between RTKs and the Hippo pathway.

RTK Subfamily (Inhibitor)	Main Finding	Ref.
*EGFR* *(gefitinib, erlotinib)*	EGFR-RAS-MAPK-Ajuba cascade inhibits SAV/LATS	[77]
	EGFR-PI3K-PDK1 inhibits LATS and regulates YAP	[78,79]
	Direct regulatory relationship between YAP activity and EGFR expression in cancer cells	[83,84,85,86]
	Positive feedback loop between YAP and EGFR	[87,88]
	YAP and ERBB4 co-localization and co-transcriptional activity	[91,92,93]
*PDGFR* *(Imatinib, Ponatinib)*	PDGFR triggers Src Family of kinases to phosphorylate YAP and activates it	[95]
	YAP increases PDGFR expression	[96]
*IR* *(Linsitinib,* *NT157)*	IR-PI3K-PDK1 Warts inhibition and Yorkie activation to induce cell proliferation	[97,98]
	IRS1 is a transcriptional target of YAP	[99]
	TAZ regulates IRS2 in liver cancer	[100]
*NTRK* *(entrectinib)*	NTRK1 regulates YAP through LATS inhibition	[102]
*c-MET* *(Crizotinib, Cabozantinib)*	HGF/MET mediated YAP/HIF-1 interaction induces expression of pluripotency master genes	[106]
	HGF-ITCH causes LATS degradation and EMT induction	[108,109]
*EPHR* *(ALW-II-41-27, Tesevatinib)*	EphA2 activates YAP/TAZ through Rho GTPase and Rock to induce glutamine metabolism	[114]
	Several Eph receptors regulate YAP activity	[115]
	EphB2 and TAZ nuclear co-localization and co-transcriptional activity	[116]
*VEGFR* *(SU4312, Apatinib)*	Reduced MST1/2 phosphorylation by VEGFR and YAP/TAZ effect on VEGFR-induced angiogenesis	[118]
	Effect of actin cytoskeleton dynamics on YAP/TAZ through VEGFR2-SFKs-Rho GTPase	[119]
*Tie* *(Altiratinib)*	YAP1/STAT3 complex regulate ANG2 expression promoting angiogenesis	[121]
	YAP-dependent expression of ANG2 is regulated by cellular confluency	[122]
	YAP/TAZ knockdown decreases Tie2 expression and blocks vascular formation	[123]
*FGFR* *(erdafitinib, Infigratinib)*	FGF2-SAPK/JNK MAP kinase signaling downregulates TAZ	[124]
	YAP/TBX5 complex controls FGFR1, -2, and -4 expression and FGF5 reduces LATS cellular levels	[126]
	FGF1/FGFR3, MAPK/ERK mediated, increase of ETV5 elevates TAZ activity	[127]
	Different doses of FGF have different effects on hippo and cause distinct outcomes in lens cells	[128]
	FGFR4 mediated breast cancer cell MST1/2 resistance	[129]
	Direct phosphorylation of YAP by FGFR, RET, and MERTK receptors	[130]
*ALK* *(crizotinib, brigatinib)*	ALK inhibits LATS and activates YAP to drive tumorigenesis phenotype	[132]
